# Etomidate in the control of severe Cushing's syndrome by neuroendocrine carcinoma

**DOI:** 10.1002/ccr3.1478

**Published:** 2018-03-12

**Authors:** Alfredo Adolfo Reza‐Albarrán, Gerson Geovany Andino Ríos, Laura Gabriela Gómez Herrera

**Affiliations:** ^1^ Department of Endocrinology and Metabolism Instituto Nacional de Ciencias Médicas y Nutrición Salvador Zubirán Vasco de Quiroga 15 Tlalpan, México City 14000 México

**Keywords:** Ectopic Cushing's syndrome, etomidate, metastatic carcinoma, octreoscan

## Abstract

Etomidate is a very effective drug in severe Cushing's syndrome that is refractory to ketoconazol. Control of the serum cortisol levels in ectopic Cushing's syndrome can be obtained with infusion rates much lower than those used in anesthesia, without respiratory side effects.

We report the case of a 51‐year‐old female with Cushing's syndrome due to ectopic ACTH secretion caused by a pancreatic tumor with a liver metastatic lesion (Fig. 1). She underwent distal pancreatectomy in 2015 by an ACTH‐secreting neuroendocrine tumor, with good initial response. In 2016, the laboratory results were morning serum cortisol: 12.72 *μ*g/dL, 24‐h urinary‐free cortisol: 105.60 *μ*g/24 h, ACTH: 163 pg/mL, FSH: 77.31 mIU/mL, LH: 68.80 mIU/mL, and testosterone: 1.09 ng/mL (0.1–0.75). Subsequently, new hepatic metastases (Fig. 2) were evident. In April 2017, she had the following tests: 24‐h urinary‐free cortisol (chemiluminescent without extraction): 3509.45 *μ*g/24 h (58–403), morning serum cortisol: 58.73 *μ*g/dL (6.7–22.6), ACTH: 1060 pg/mL (10–100), TSH: 0.41 mIU/L (0.3–5), free T4: 0.91 ng/dL (0.63–1.34), FSH: 0.50 mIU/mL (16.74–113.59), LH: 0.36 mIU/mL (10.87–58.64), estradiol: 20.0 pg/mL (<620), and chromogranin A: 15.0 nmol/L (≤3); the low LH and FSH values were explained by the severe hypercortisolism. The patient was being treated with ketoconazole 1000 mg/day in order to reduce endogenous hypercortisolism in the event of bilateral adrenalectomy (Fig. 3) as a definitive treatment method and, later, the treatment of liver metastases through chemoembolization. The first days of April 2017, the patient entered the emergency room for a sepsis of intestinal origin. As control of cortisol levels was not acceptable despite high doses of ketoconazole, it was decided to initiate therapy with infused etomidate to attempt for the reduction in the possibility of associated complications to trans‐ and postoperative bilateral adrenalectomy. The initial dose was 0.04 mg/kg/h; daily measurements of serum cortisol, and 24‐h urinary‐free cortisol were performed (Figs. 4 and 5). The patient was maintained with the infusion of etomidate for 2 weeks. The dose was gradually decreased according to the daily cortisol results; a dose of 0.01 mg/kg/h was employed during the last 4 days of use. The day before surgery, the infusion was stopped; 8 h before surgery, hydrocortisone replacement therapy was started: 100 mg IV every 8 h. No effect on respiratory function was observed. The general state of the patient improved markedly, and control of the septic process was achieved with relative ease. Bilateral adrenalectomy was performed without any complications. The patient is currently being treated with hydrocortisone and fludrocortisone; treatment of liver metastases is pending.

**Figure 1:**
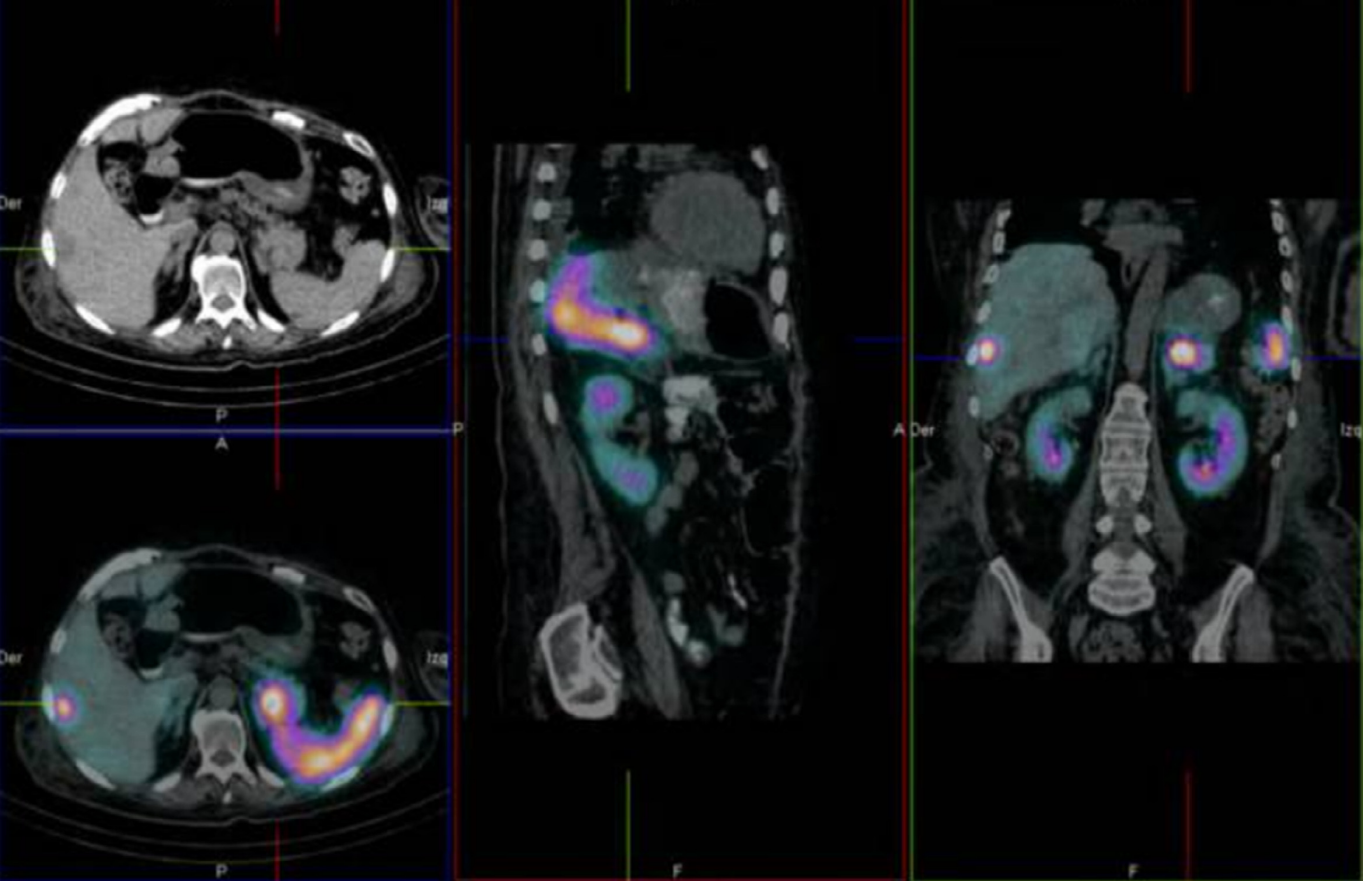
A SPECT‐CT shows a pancreatic tumor with a metastatic liver lesion.

**Figure 2:**
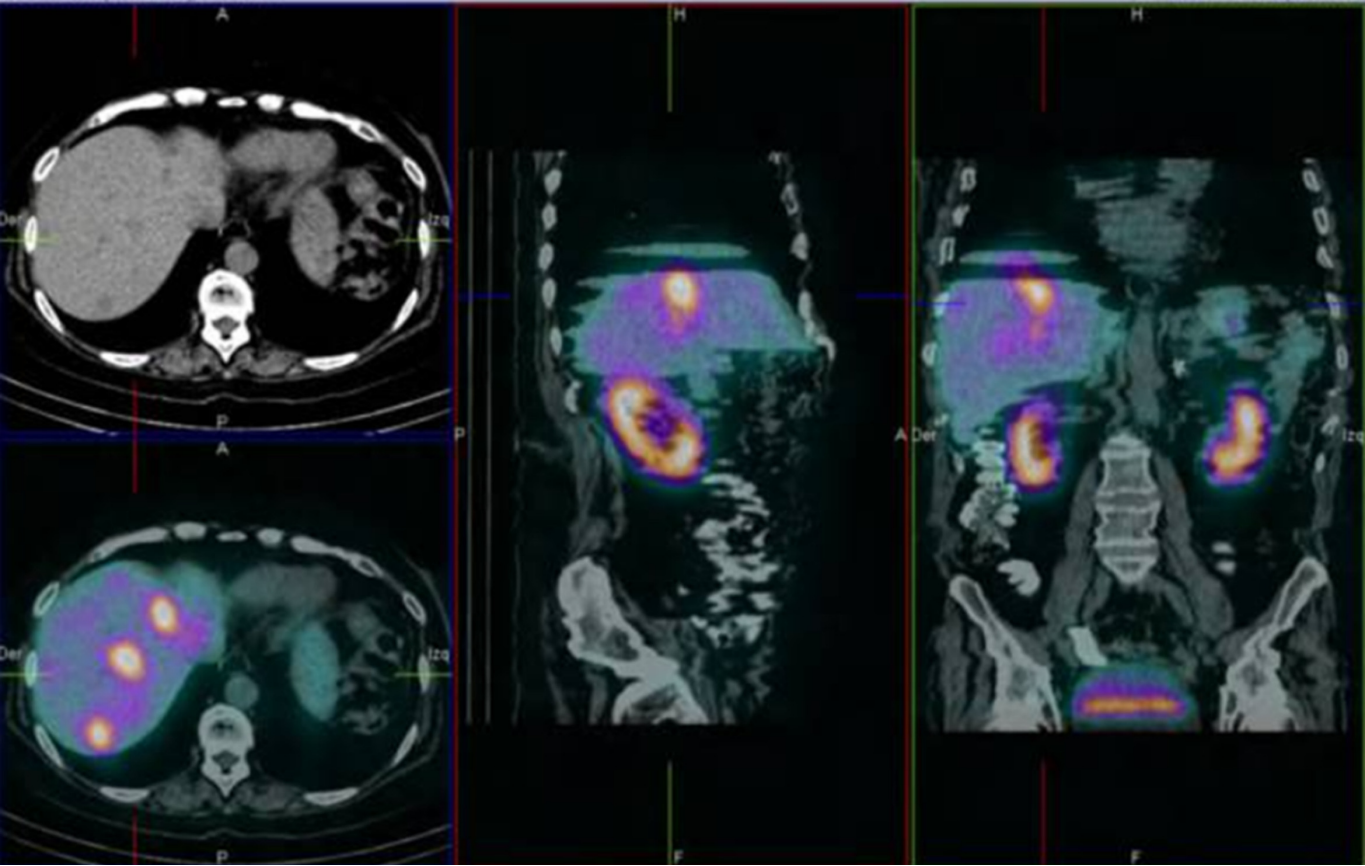
SPECT‐CT showing liver metastases secondary to neuroendocrine ACTH‐secreting pancreatic tumor.

**Figure 3:**
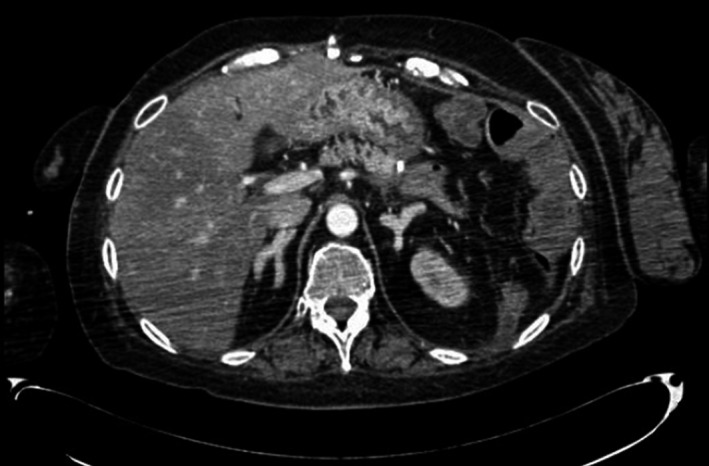
Abdominal CT scan showing bilateral adrenal hyperplasia secondary to the chronic stimulatory effect of ectopic ACTH secretion.

**Figure 4:**
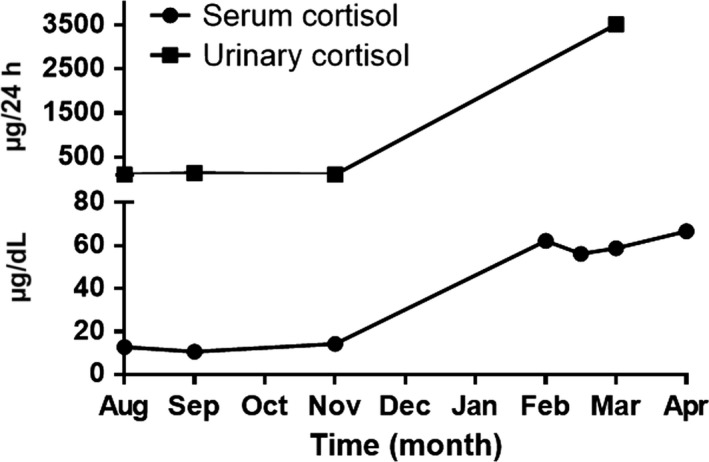
Twenty‐four hour urinary‐free cortisol levels and morning serum cortisol before etomidate infusion.

**Figure 5:**
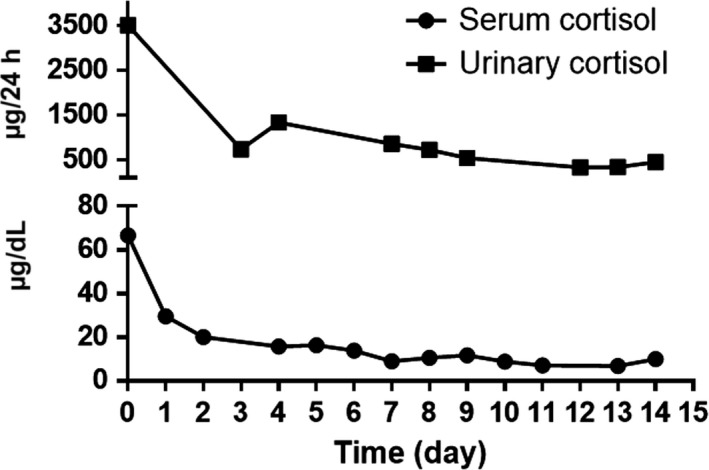
Twenty‐four hour urinary‐free cortisol levels and morning serum cortisol during etomidate infusion.

Ectopic Cushing's syndrome is the second most frequent cause of ACTH‐dependent hypercortisolism (15–20%) 1. Regardless of the origin of endogenous hypercortisolism, the treatment of choice is the surgical treatment. Pharmacological treatment is indicated in case of relapse or persistence of endogenous hypercortisolism 2. Although ketoconazole is generally the drug of choice for the control of adrenal synthesis, the therapeutic effect is often limited and associated with side effects. Etomidate is a potent inhibitor of the enzyme 11‐beta‐hydroxylase, which catalyzes the conversion of 11‐deoxycortisol to cortisol 3. There are few case reports of the use of etomidate in the control of severe hypercortisolism; generally, their role is the rapid control of acute or life‐threatening complications; in this case, its use was indicated in the absence of adequate response to the use of ketoconazole in the control of severe hypercortisolism and its consequent catabolic effects; the biochemical control would also be useful for the surgical intervention to be performed when hypercortisolism is controlled. Some reports have described the use of etomidate together with hydrocortisone under a “block‐substitution” scheme 4. During the infusion with etomidate electrolytic alterations such as hypokalemia (2.53 mmol/L), hypocalcemia (7.1 mg/dL), hypophosphatemia (1.3 mg/dL), and hypomagnesemia (1.3 mg/dL) were presented, which were corrected immediately after the gradual reduction in the dose of etomidate and which we considered to be secondary to renal tubular damage associated with etomidate drug 5. In this patient with life‐threatening conditions associated with Cushing's syndrome, etomidate was an effective therapeutic measure. We suggest that the initial dose should be 0.04 mg/kg/h, daily monitoring of 24‐h urinary cortisol and cortisol levels is recommendable and that the dose of the drug should be gradually decreased according to the daily measurements of metabolites; the first decrease from 0.04 to 0.03 mg/kg/h may have to be performed within 48 h of drug initiation; and the final dose may be as low as 0.01 mg/kg/h.

## Authorship

AARA: treated the patient with etomidate and wrote the manuscript. GGAR: participated in the writing of the manuscript and uploading of image figures. LGGH: participated in the writing of manuscript and follow‐up of the patient.

## Conflict of Interest

There is no conflict of interest that could be perceived as prejudicing the impartiality of the research reported.
